# Supplementary material for: Time-Lagged Multidimensional Pattern Connectivity (TL-MDPC): An EEG/MEG pattern transformation based functional connectivity metric
Source: Neuroimage. 2023 Apr 15;270:119958. doi: 10.1016/j.neuroimage.2023.119958 (PMC10030313; doi:10.1016/j.neuroimage.2023.119958)
Supplement: Supplementary file 1 [file mmc1.docx]

Supplementary

|  | **TL-MDPC** | **TL-UDC** |
| --- | --- | --- |
|  | \| **SD** \| **LD** \| **Comparison** \| \| --- \| --- \| --- \| | \| **SD** \| **LD** \| **Comparison** \| \| --- \| --- \| --- \| |
| **lATL-rATL** | 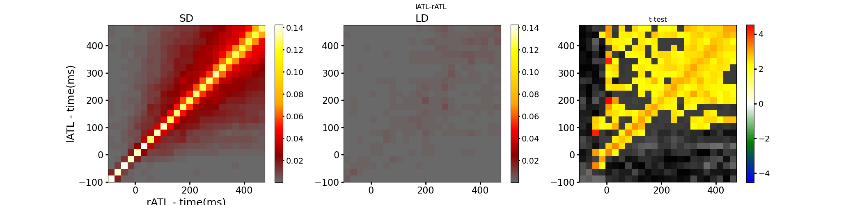 | 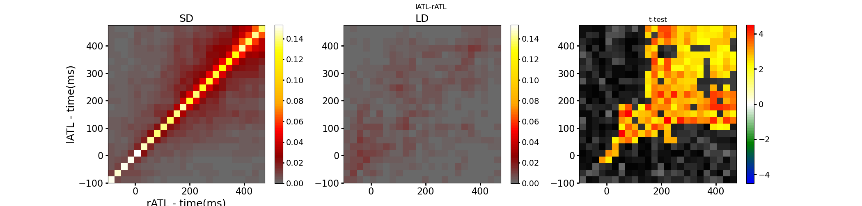 |
| **lATL-PTC** | 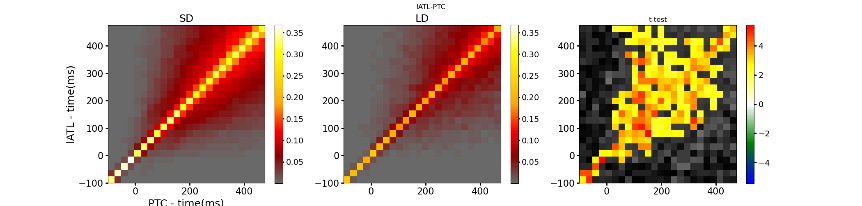 | 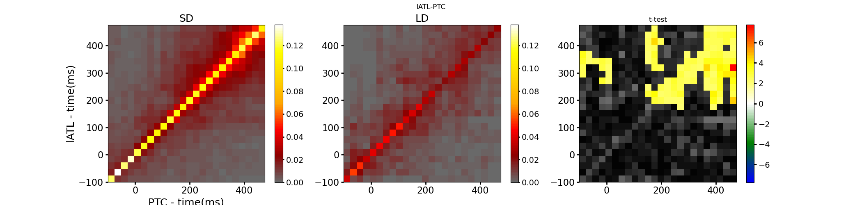 |
| **lATL-IFG** | 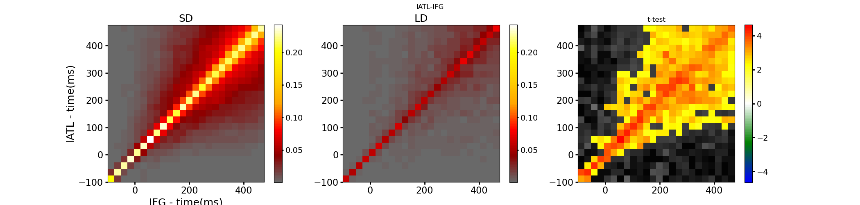 | 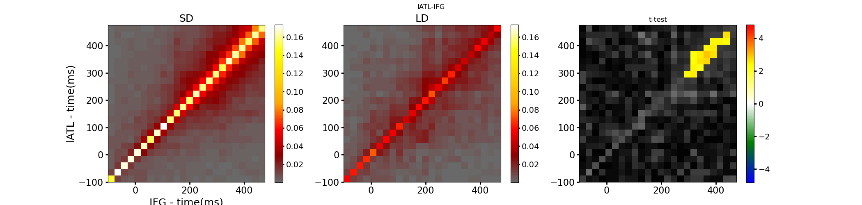 |
| **lATL-AG** | 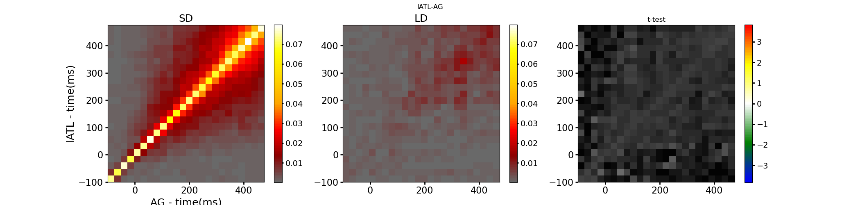 | 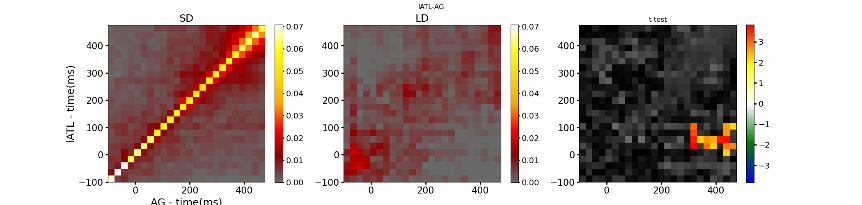 |
| **lATL-PVA** | 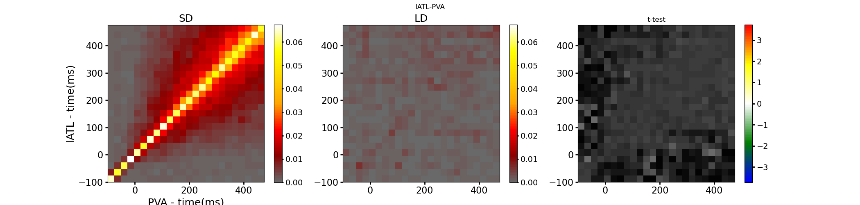 | 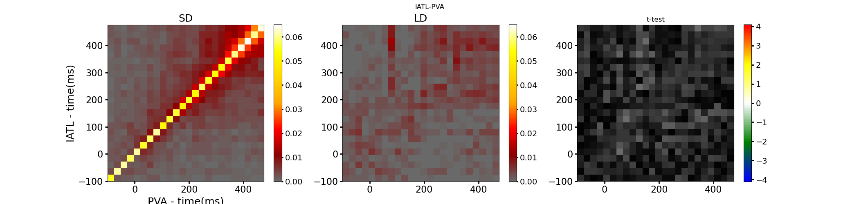 |
| **rATL-PTC** | 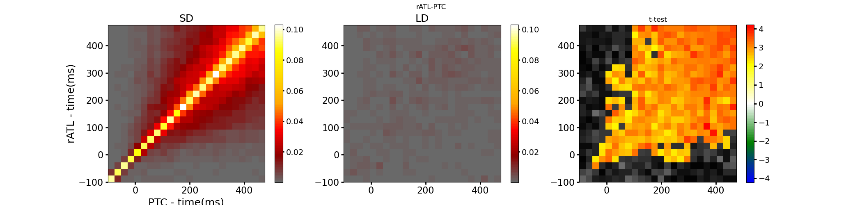 | 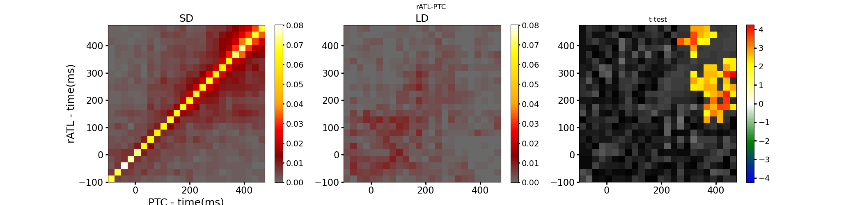 |
| **rATL-IFG** | 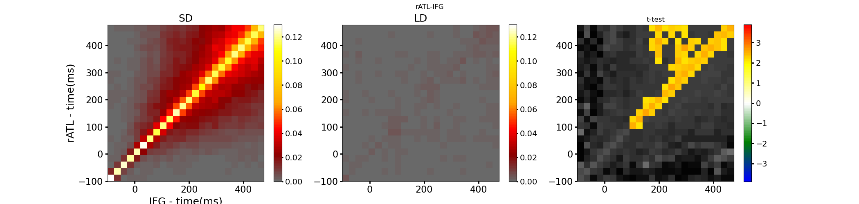 | 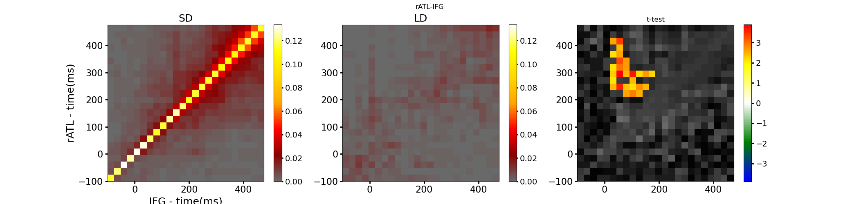 |
| **rATL-AG** | 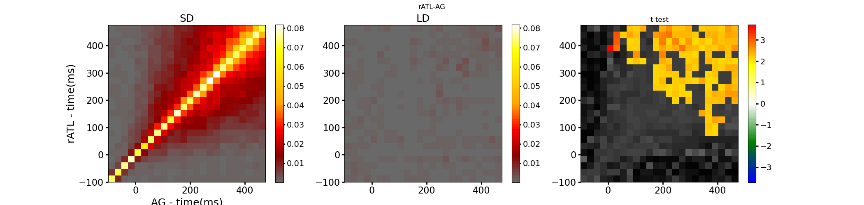 | 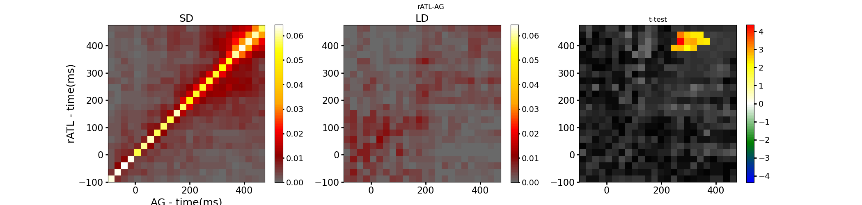 |
| **rATL-PVA** | 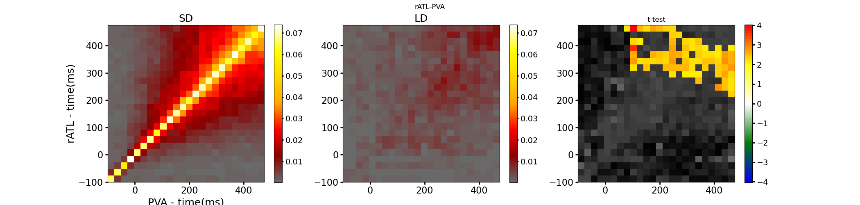 | 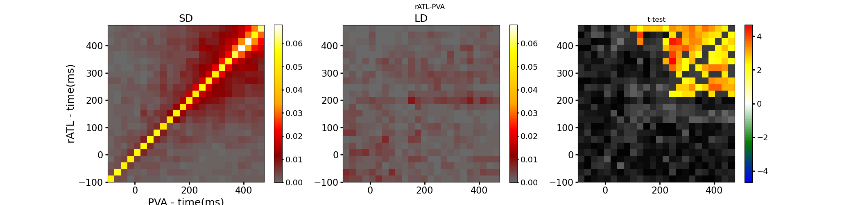 |
| **PTC-IFG** | 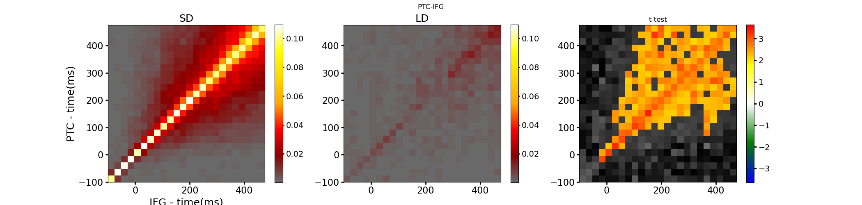 | 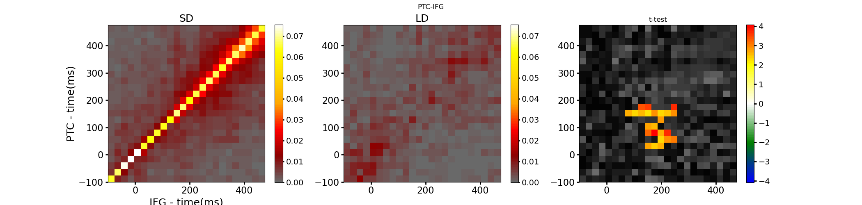 |
| **PTC-AG** | 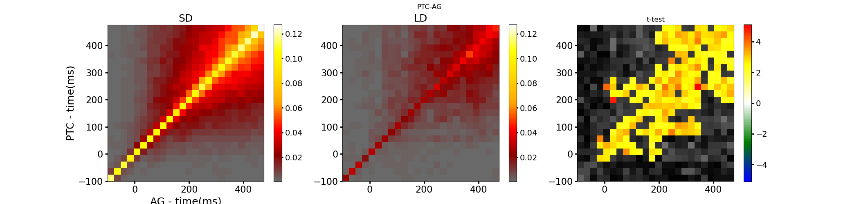 | 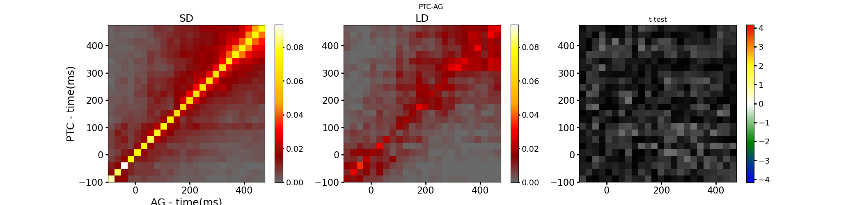 |
| **PTC-PVA** | 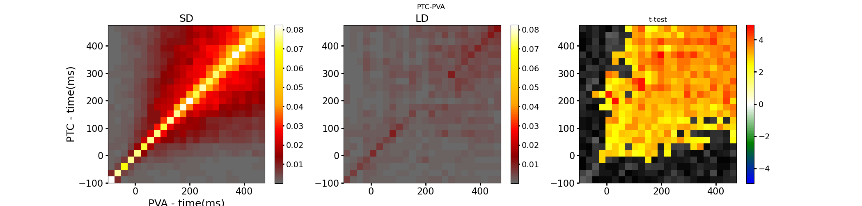 | 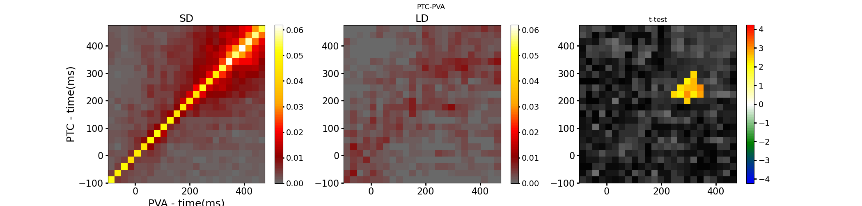 |
| **IFG-AG** | 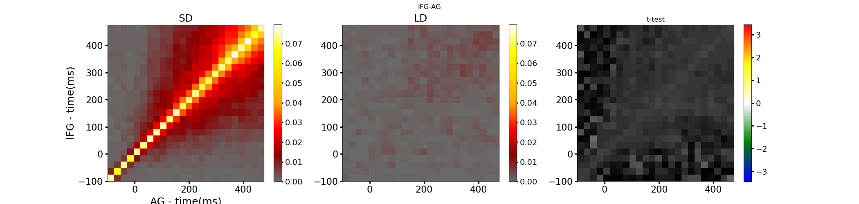 | 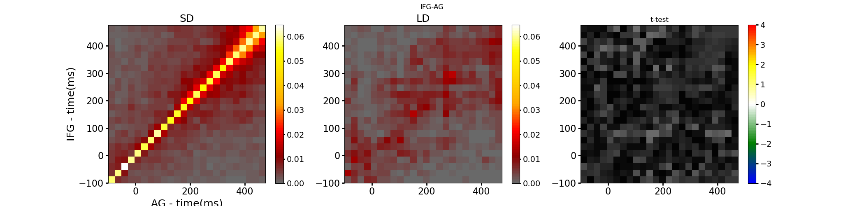 |
| **IFG-PVA** | 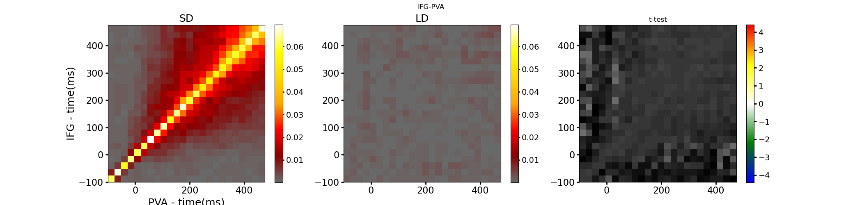 | 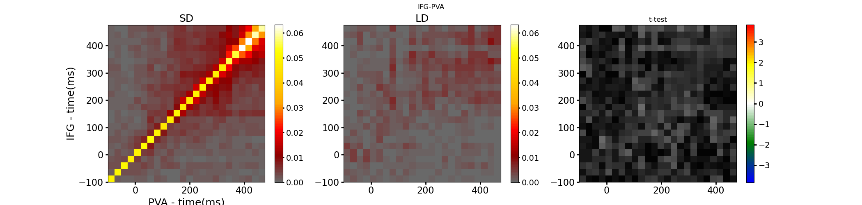 |
| **AG-PVA** | 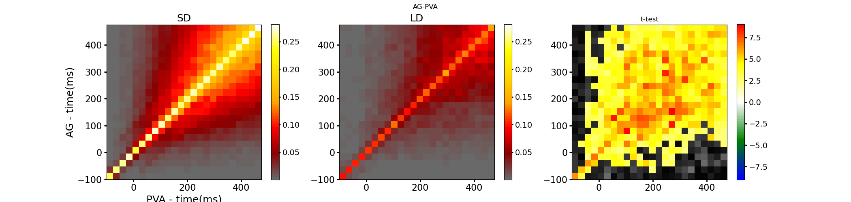 | 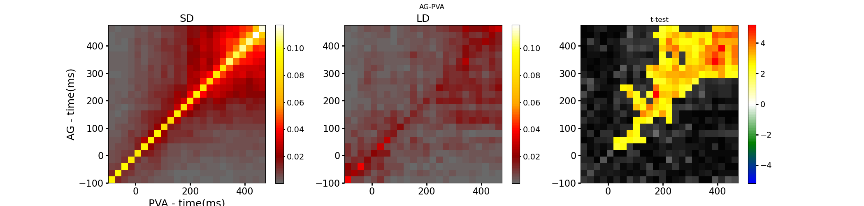 |


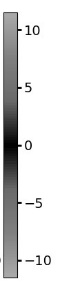
Figure S1- Representation of all TTMs for semantic decision (SD) and lexical decision (LD) tasks, and their comparison, using TL-MDPC (left column) and TL-UDC approach (right column). TTMs for SD and LD are averaged across participants, and comparison was performed using cluster-based permutation test with alpha-level=0.05. All significant contrasts show greater connectivity for SD using MD. The size of all clusters shown here is greater than 2% of TTMs size (24*24). The gray-scale colorbar indicates non-significant t-values (this color bar is the same across all Figures).

|  | **TL-MDPC – TL-UDC comparision** |
| --- | --- |
|  | \| **TL-MDPC** \| **TL-UDC** \| **Comparison** \| \| --- \| --- \| --- \| |
| **lATL-rATL** | 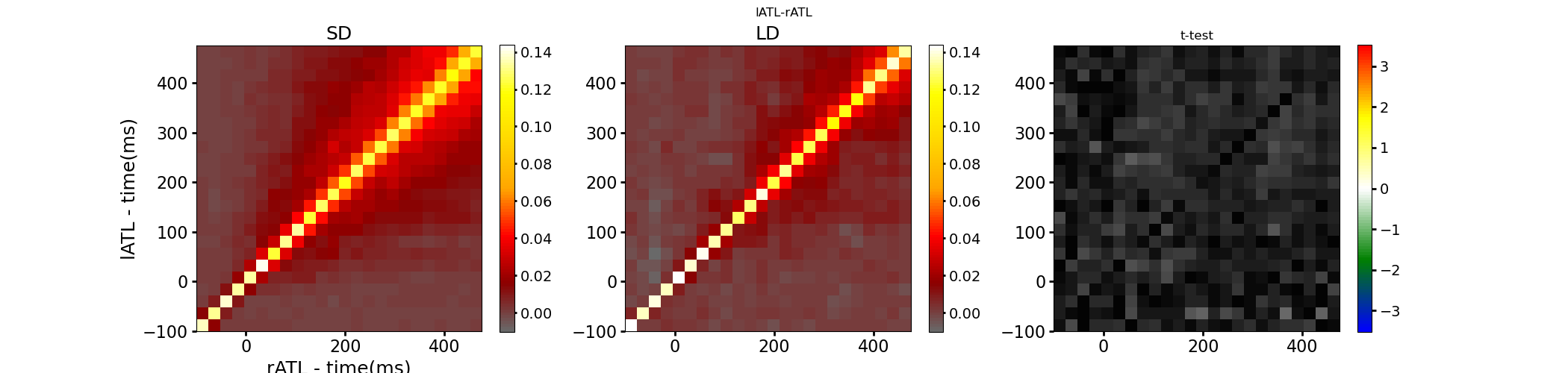 |
| **lATL-PTC** | 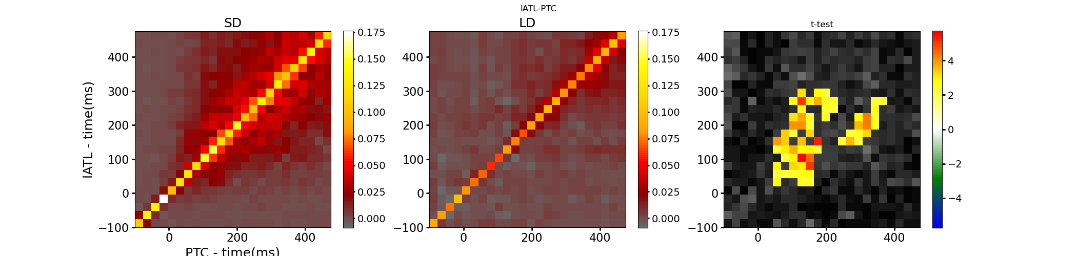 |
| **lATL-IFG** | 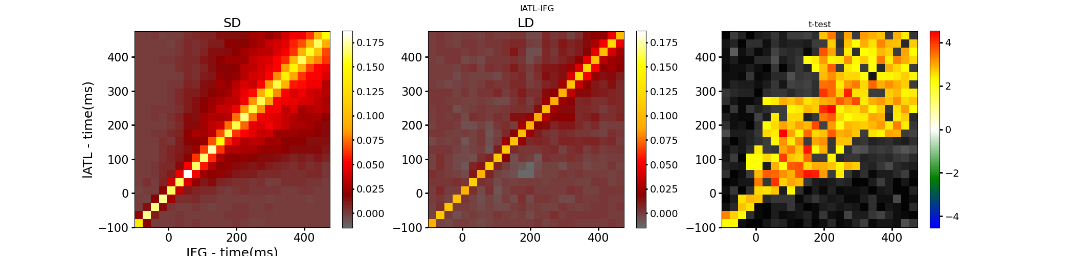 |
| **lATL-AG** | 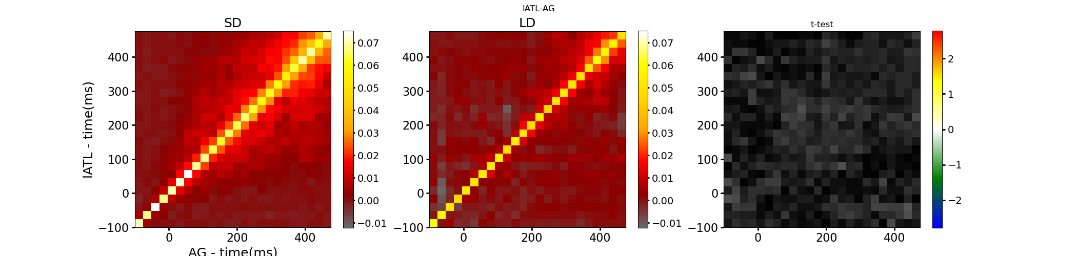 |
| **lATL-PVA** | 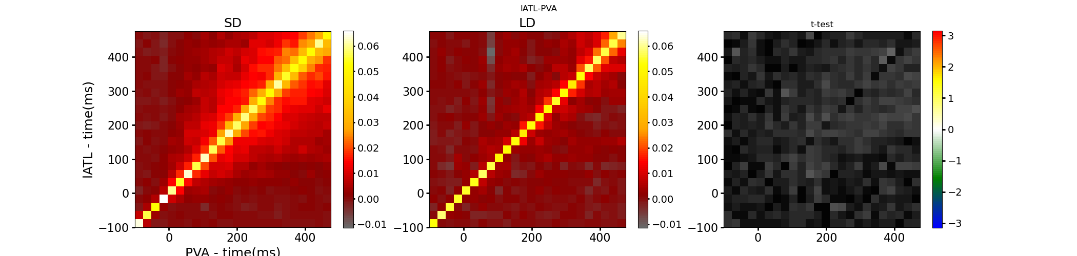 |
| **rATL-PTC** | 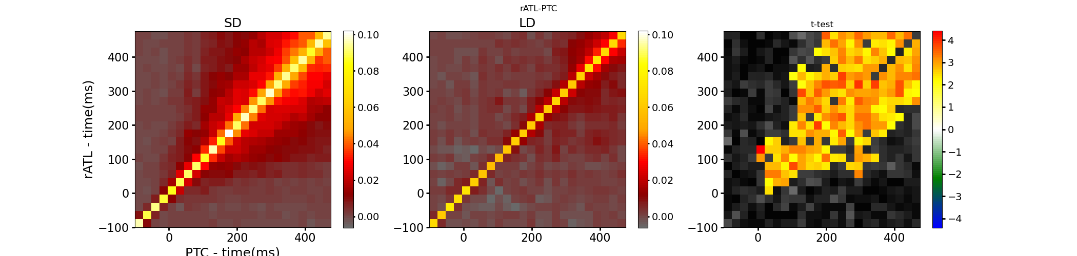 |
| **rATL-IFG** | 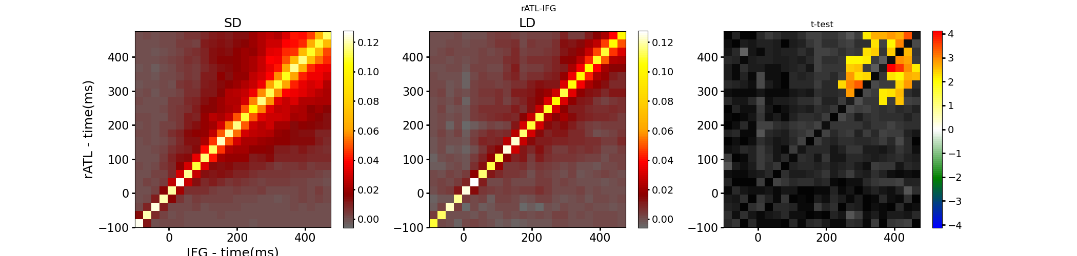 |
| **rATL-AG** | 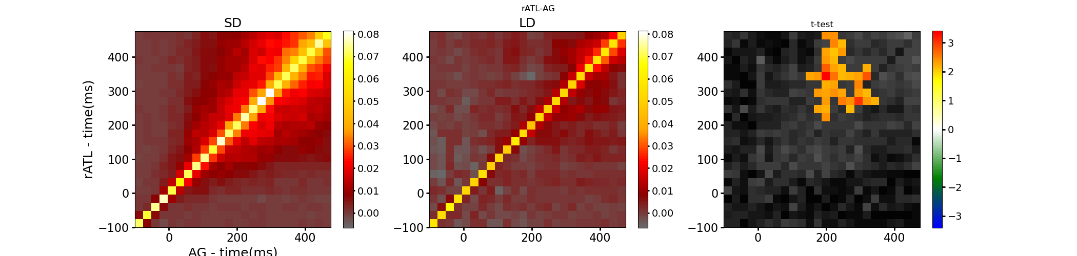 |
| **rATL-PVA** | 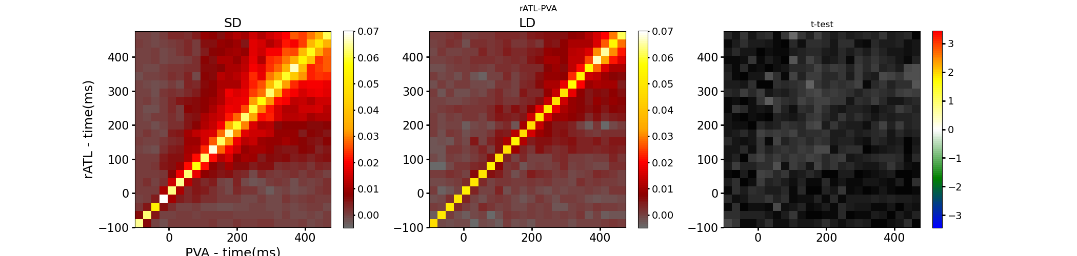 |
| **PTC-IFG** | 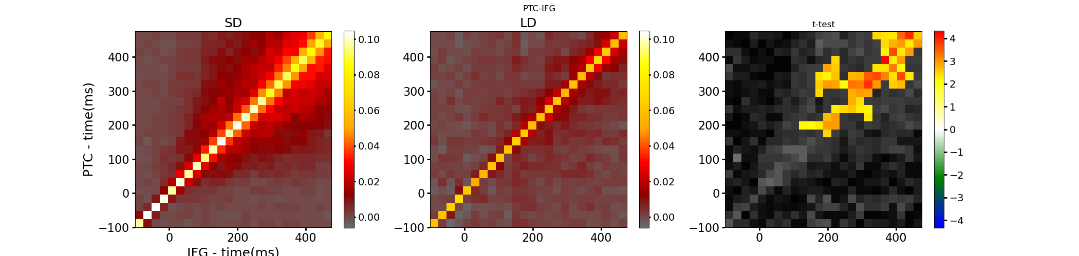 |
| **PTC-AG** | 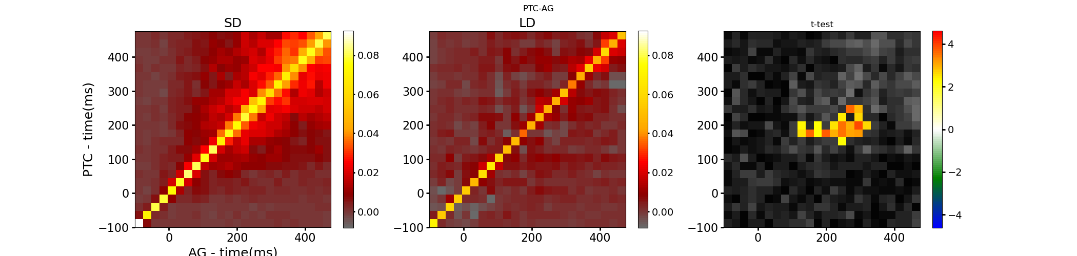 |
| **PTC-PVA** | 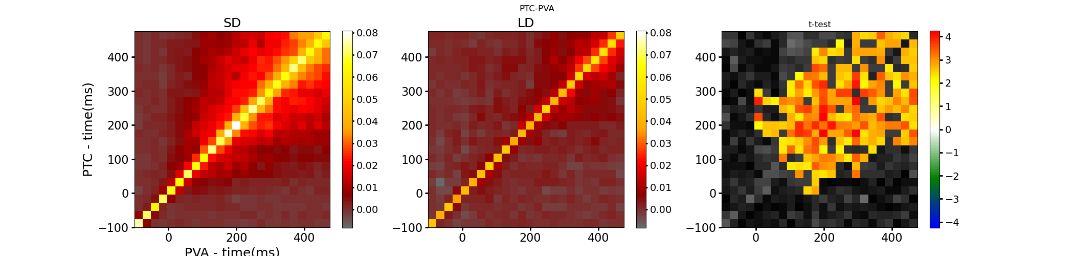 |
| **IFG-AG** | 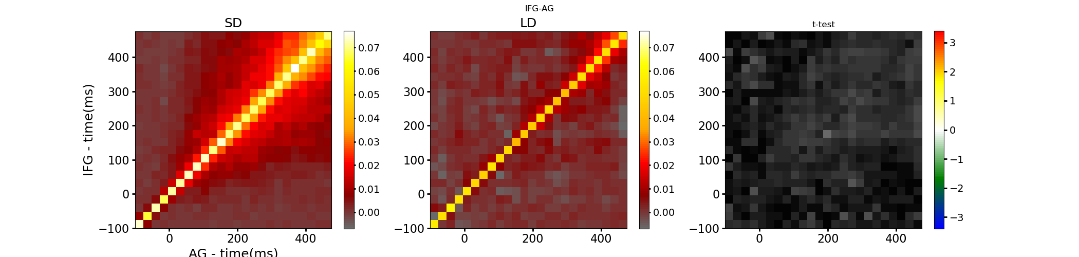 |
| **IFG-PVA** | 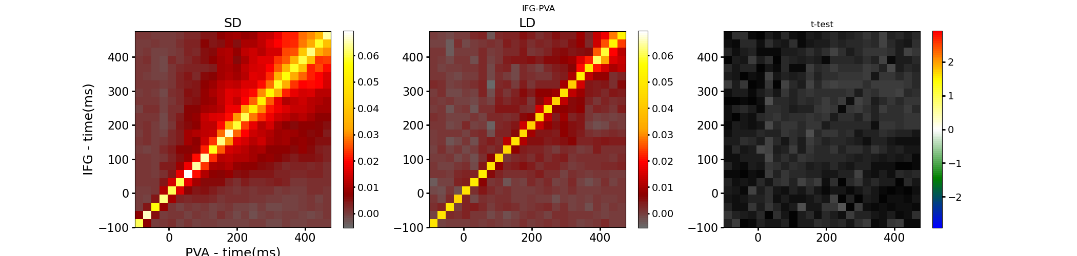 |
| **AG-PVA** | 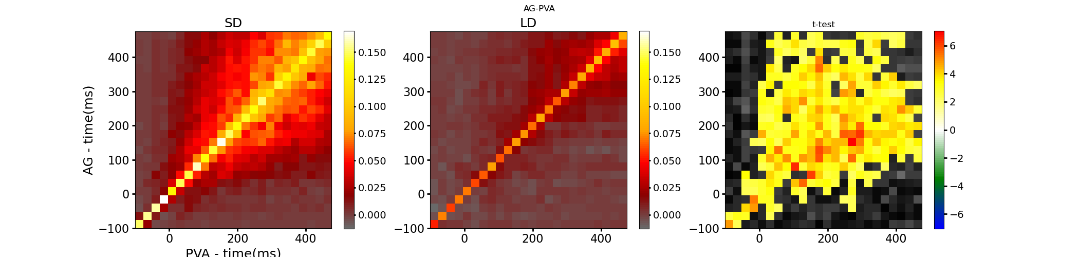 |


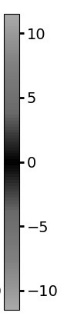


Figure S2- Direct comparison of TL-MDPC and TL-UDC of all TTMs for the methods comparisons. The left column shows the average of task modulations (semantic decision (SD) - lexical decision (LD) using TL-MTPC, the middle column shows average of task modulations (SD>LD) using TL-UDC, and the right column shows the contrast between these SD and LD results for the two methods (TL-MDPC, TL-UDC) using a cluster-based permutation test with alpha-level=0.05. All significant contrasts show greater connectivity for MDPC compared to UDC. The size of all clusters shown here is greater than 2% of TTMs size (24*24). The gray-scale colorbar indicates non-significant t-values (this color bar is the same across all Figures).
